# A Validated Isocratic HPLC–UV Method for the Simultaneous Quantification of Corilagin and Geraniin in *Geranium wilfordii* Maxim. Extract

**DOI:** 10.3390/molecules31010031

**Published:** 2025-12-22

**Authors:** Jung-Min Kim, Kun-Ho Song, Yong-Seok Choi, Cheon-Kwang Ko, Bong-Seop Lee

**Affiliations:** 1 Department of Chemical Engineering, Kangwon National University, Chuncheon 24341, Gangwon, Republic of Korea; wjdals9725@kangwon.ac.kr; 2 Newgen Healthcare Co., Ltd., 56 Soyanggang-ro, Chuncheon 24232, Gangwon, Republic of Korea; 3 University-Industry Cooperation Center, University-Industry Cooperation Foundation, Kangwon National University, Chuncheon 24341, Gangwon, Republic of Korea; 4 Gangwon Regional Institute of Industrial Advancement, Kangwon National University, Chuncheon 24341, Gangwon, Republic of Korea

**Keywords:** corilagin, geraniin, *Geranium wilfordii* Maxim., HPLC analysis, method validation, quality control

## Abstract

*Geranium wilfordii* Maxim. is a traditional medicinal plant rich in ellagitannins such as corilagin (CG) and geraniin (GR), which possess antioxidant and anti-inflammatory properties. However, accurate quantification of CG and GR in complex herbal matrices is hindered by co-eluting impurities and poor UV resolution. Here, we developed and validated a simple isocratic HPLC–UV method for their simultaneous determination in *G. wilfordii* extract. Separation was achieved on a Polaris 3 C18-A column (250 mm × 4.6 mm, 3 µm) using acetonitrile/0.2% formic acid in water (11:89, *v*/*v*) with UV detection at 270 nm. The method showed excellent linearity (25–300 µg/mL, R^2^ > 0.995), precision (RSD < 2.7%), accuracy (recovery 99.5–101.2%), and low detection limits (<3 µg/mL). Previous approaches have relied on gradient HPLC or MS-based techniques, often requiring long run times, costly instrumentation, or additional purification (e.g., HSCCC). In contrast, this study demonstrates a validated isocratic method that enables baseline separation and simultaneous quantification of CG and GR in a single run. This robust and simplified analytical strategy provides a practical tool for routine quality control and phytochemical standardization, with potential applications across pharmaceutical, food, and cosmetic industries.

## 1. Introduction

*Geranium wilfordii* Maxim. (Geraniaceae) is widely distributed across East Asia, including China, Japan, and South Korea [1]. The plant has been used in traditional Chinese medicine for over 600 years to manage disorders such as arthritis, dysentery, and diarrhea [2,3]. Despite this long history of traditional use, comprehensive pharmacological investigations remain limited, underscoring the need for further characterization of its bioactive constituents. Accordingly, recent studies have increasingly focused on the phytochemistry and therapeutic potential of *G. wilfordii*, aiming to identify its diverse chemical components and clarify the specific molecules responsible for its biological effects. To date, more than 90 compounds have been identified, including tannins, flavonoids, flavonol glycosides, organic acids, and steroids [4,5,6]. Among these constituents, hydrolyzable tannins—particularly ellagitannins—have attracted considerable attention due to their proposed roles in mediating the plant’s biological activities [7].

**Figure 1:**
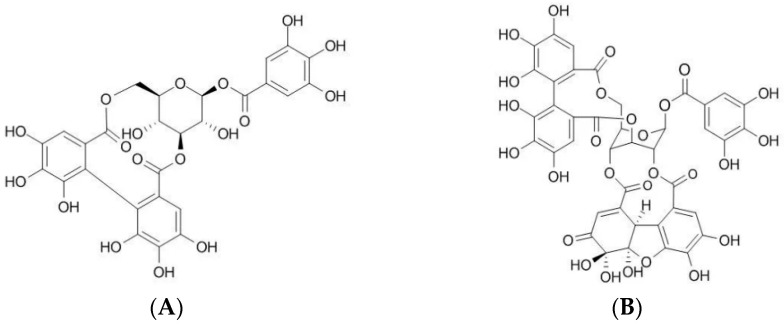
Chemical structures of (**A**) corilagin and (**B**) geraniin.

Corilagin (CG) and geraniin (GR) are the major ellagitannins present in *G. wilfordii* and have been associated with various biological effects [8]. The chemical structures of CG and GR are shown in Figure 1. CG has been shown to improve rheumatoid arthritis by downregulating nuclear factor-kappa B (NF-κB) and mitogen-activated protein kinase (MAPK) signaling pathways [9] and also demonstrates anti-allergic effects [10]. GR exhibits anti-hyperglycemic [11], osteoprotective [12], anti-hypertensive [13], hepatoprotective [14]. Additionally, both CG and GR have demonstrated potential as antioxidants [15], anti-inflammatory [16], anti-diabetic [17], anti-hypercholesterolemia effects [18]. Owing to these diverse pharmacological activities, CG and GR serve as key molecular markers and biologically relevant targets for quantitative analysis in *G. wilfordii*.

Accurate quantification of CG and GR is essential for biological evaluation and for establishing reliable quality-control standards for *G. wilfordii* extracts. Although MS-based analytical methods such as UPLC-QQQ-MS [19] and HPLC-ESI-MS [20] offer high sensitivity, they typically require extensive sample preparation and may not consistently achieve adequate chromatographic resolution of the two ellagitannins. More accessible UV- or PDA-based HPLC approaches frequently encounter insufficient separation in crude extracts, often necessitating additional purification steps such as high-speed counter-current chromatography (HSCCC) [21]. These limitations—including long run times, high operational costs, and poor chromatographic resolution—highlight the need for a simplified, reproducible, and cost-effective analytical method capable of resolving both analytes under isocratic conditions.

To address this need, an isocratic HPLC–UV method was developed and validated in this study that enables baseline separation and simultaneous quantification of CG and GR in *G. wilfordii* extract. While reversed-phase HPLC has been previously applied for analyzing these compounds, the present study provides a fully validated and operationally accessible isocratic method optimized for routine use. The method employs a simple mobile-phase composition and minimal sample preparation, supporting its strong applicability to phytochemical analysis and quality-control workflows.

## 2. Results and Discussion

Reversed-phase chromatography is the most widely applied mode in HPLC and allows flexible adjustment of the mobile phase through modification of the organic solvent, pH, and operational parameters such as flow rate and temperature [22]. However, when operated under highly aqueous conditions, conventional reversed-phase columns are prone to phase collapse, resulting in reduced separation efficiency and poor reproducibility for polar analytes [23].

To mitigate this issue, specialized stationary phases such as alkyl-bonded columns have been introduced, although their chromatographic selectivity often differs markedly from that of conventional C18 materials, limiting their broader applicability [24]. Therefore, C18 columns engineered to tolerate 100% aqueous mobile phases were selected in this study. These columns enabled stable operation under fully aqueous conditions and provided reliable separation of CG and GR from *G. wilfordii* extract, which contains abundant polar constituents.

### 2.1. Method Development

**Figure 2:**
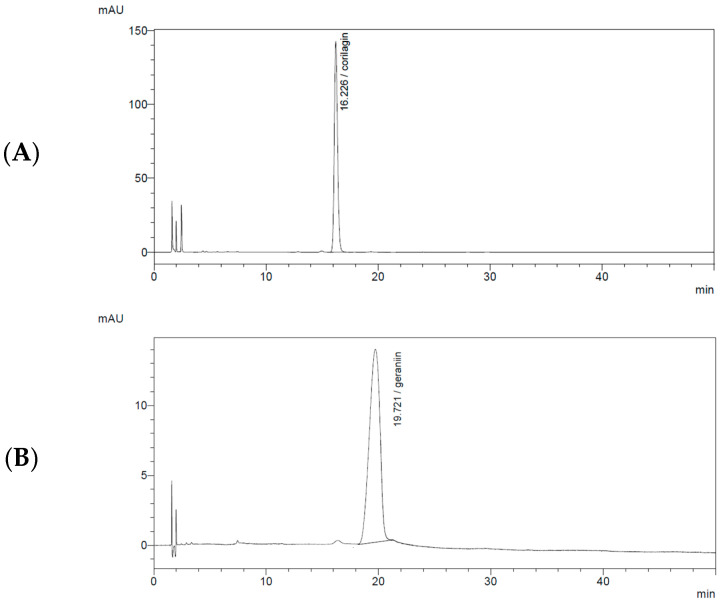

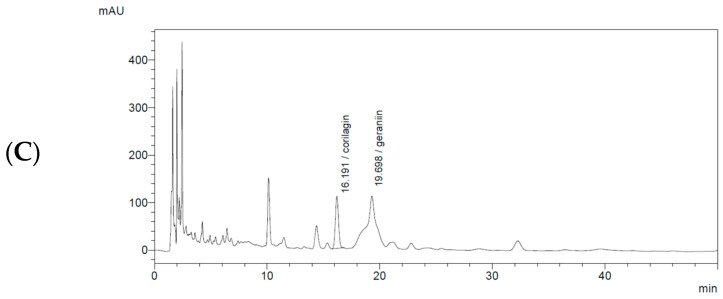
HPLC chromatograms of corilagin and geraniin in standard solution and *Geranium wilfordii* extract using an XDB-C18 column (150 mm × 4.6 mm, 3.5 µm) with a mobile phase of 0.2% formic acid in water (90%):acetonitrile (10%). (**A**) Corilagin standard solution; (**B**) Geraniin standard solution; (**C**) *Geranium wilfordii* extract.

Preliminary experiments were performed using an XDB-C18 reversed-phase column (150 mm × 4.6 mm, 3.5 µm; Agilent, Santa Clara, CA, USA), and several mobile-phase compositions were evaluated to optimize the separation of CG and GR from other constituents in *G. wilfordii* extract. Chromatographic performance was assessed using parameters such as tailing factor, resolution, and selectivity. The XDB-C18 column was operated with a mobile phase of 0.2% formic acid in water (90%) and acetonitrile (10%) at a flow rate of 1.0 mL/min. Although this setup provided excellent separation for the standard solutions, satisfactory resolution and reproducibility could not be consistently achieved for CG and GR in the complex extract matrix. In particular, the GR peak overlapped with adjacent constituents and exhibited significant peak broadening (Figure 2).

Although corilagin and geraniin can be readily separated in standard solutions, chromatographic separation becomes significantly more challenging in *G. wilfordii* extract due to the presence of multiple unknown co-eluting components. Therefore, method optimization focused on resolving CG and GR from surrounding matrix peaks rather than on their mutual separation alone.

To improve peak resolution, the proportion of acetonitrile (ACN) in the mobile phase was initially reduced. However, this adjustment resulted in pronounced peak tailing and poor reproducibility across repeated injections. Methanol was subsequently evaluated as an alternative organic modifier; however, the geraniin peak was not detected. This behavior is consistent with previous reports indicating that methanol can react with the hemiacetal moiety of geraniin to form methoxy-containing adducts, which often manifest as multiple or broadened peaks in HPLC chromatograms [25]. Accordingly, methanol was excluded from the mobile phase during method development.

In contrast, 20% (*v*/*v*) methanol was employed only for sample dissolution and standard preparation under mild conditions. The extract solutions were prepared immediately prior to analysis without prolonged storage or thermal exposure. Under these conditions, no peak distortion, degradation, or loss of geraniin was observed, as evidenced by stable retention times, consistent peak areas, and satisfactory validation results. Taken together, these observations indicated that more polar mobile-phase conditions were required to achieve effective separation of CG and GR in the *G. wilfordii* extract while maintaining analytical stability.

To address the limitations observed with the conventional reversed-phase column, a polar-embedded C18 column (Polaris C18-A), engineered for stability under 100% aqueous conditions, was subsequently assessed. Increasing the water content in the mobile phase improved chromatographic resolution but substantially prolonged the analysis time [26]. Therefore, a maximum run time of 40 min was set, and the acetonitrile concentration was systematically adjusted (8–12%, *v*/*v*) to achieve a resolution (R_s_) greater than 1.5 between adjacent peaks.

Two Polaris columns with different lengths—Polaris 3 C18-A (150 mm × 4.6 mm, 3 µm) and Polaris 3 C18-A (250 mm × 4.6 mm, 3 µm)—were evaluated to determine their effectiveness in resolving CG and GR from other constituents in the *G. wilfordii* extract. A series of mobile phases with varying ACN concentrations (8–12%, *v*/*v*) was tested, and the elution times and resolution values of CG and GR were assessed. All experiments were performed under consistent operating conditions: a flow rate of 1.0 mL/min, a column temperature of 30 °C, a detection wavelength of 270 nm, and a maximum run time of 40 min.

**Figure 3:**
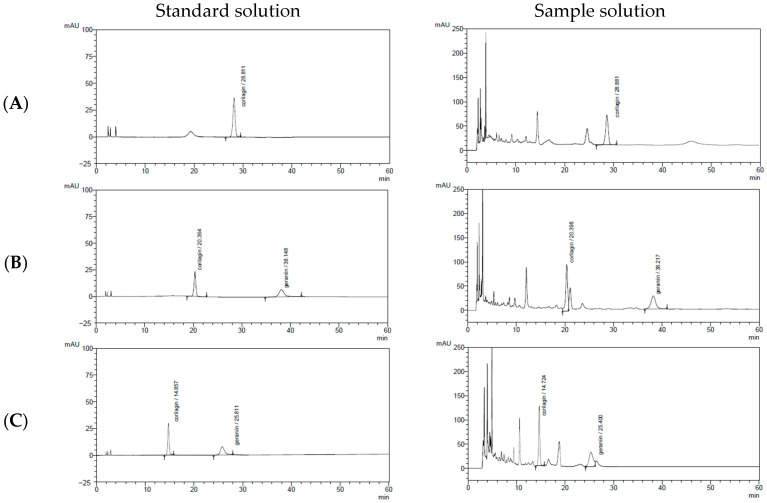
HPLC chromatograms of corilagin and geraniin in standard solution and *Geranium wilfordii* extract under different mobile phase ratios with a Polaris 3 C18-A column (150 mm × 4.6 mm). (**A**) 0.2% formic acid in water (92%):acetonitrile (8%); (**B**) 0.2% formic acid in water (91%):acetonitrile (9%); (**C**) 0.2% formic acid in water (90%):acetonitrile (10%). Arrows indicate the chromatographic peaks of corilagin and geraniin.

**Figure 4:**
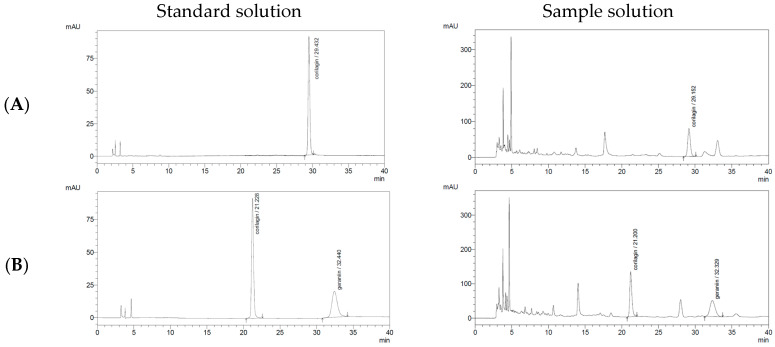

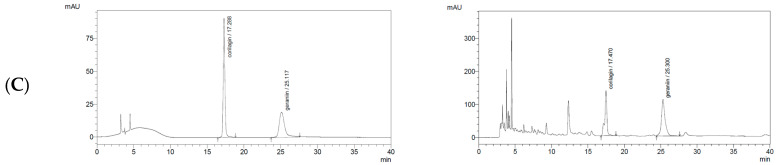
HPLC chromatograms of corilagin and geraniin in standard solution and *Geranium wilfordii* extract under different mobile phase ratios with a Polaris 3 C18-A column (250 mm × 4.6 mm). (**A**) 0.2% formic acid in water (90%): acetonitrile (10%); (**B**) 0.2% formic acid in water (89%):acetonitrile (11%); (**C**) 0.2% formic acid in water (88%):acetonitrile (12%). Arrows indicate the chromatographic peaks of corilagin and geraniin.

As shown in Figure 3 and Figure 4, increasing the column length to 250 mm markedly improved peak resolution, producing sharper and more symmetrical peaks with reduced baseline noise. Based on these comparative studies, the optimal analytical conditions for the simultaneous determination of CG and GR in *G. wilfordii* extract were established as follows: a mobile phase of acetonitrile–water (11:89, *v*/*v*) on a Polaris 3 C18-A column (250 mm × 4.6 mm, 3 µm), with a flow rate of 1.0 mL/min, a column temperature of 30 °C, and UV detection at 270 nm (Table 1).

### 2.2. Method Validation Results

#### 2.2.1. Specificity Results

**Figure 5:**
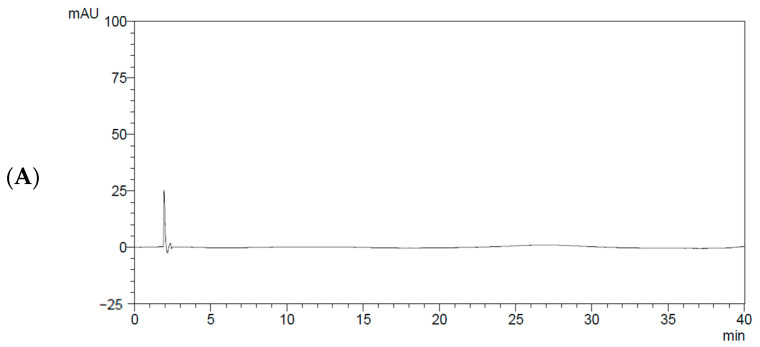

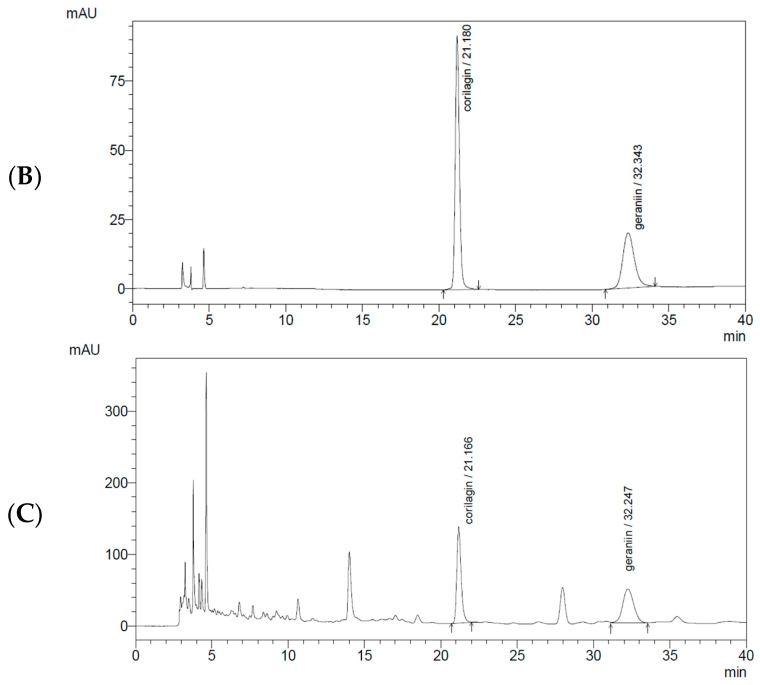
HPLC chromatograms presenting the specificity of the validated method for corilagin and geraniin. Chromatograms of (**A**) blank matrix (20% (*v*/*v*) methanol), (**B**) mixed standard solution, and (**C**) *Geranium wilfordii* extract. Arrows indicate the chromatographic peaks of corilagin and geraniin.

**Figure 6:**
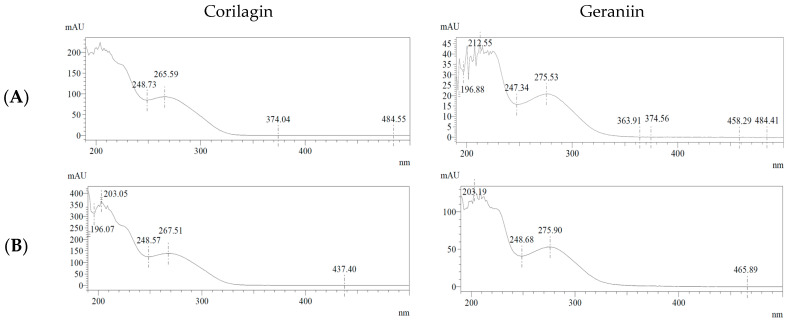
UV spectra of corilagin and geraniin. (**A**) Standard solutions of corilagin and geraniin; (**B**) *Geranium wilfordii* extract.

Specificity was evaluated through chromatographic analysis of both standard and sample solutions. Corilagin (CG) and geraniin (GR) were clearly separated, exhibiting retention times of 21.17 min and 32.25 min, respectively (Figure 5). Both analytes showed baseline-resolved peaks without interference from adjacent matrix components, as evidenced by resolution (Rs) values greater than 1.5 and tailing factors below 2. Peak purity was further confirmed using PDA-based UV spectral analysis. The UV spectra of CG and GR in the extract matched those of the corresponding standards across the entire peak profiles (Figure 6), clearly confirming peak homogeneity and demonstrating adequate method specificity.

#### 2.2.2. Linearity Results

**Figure 7:**
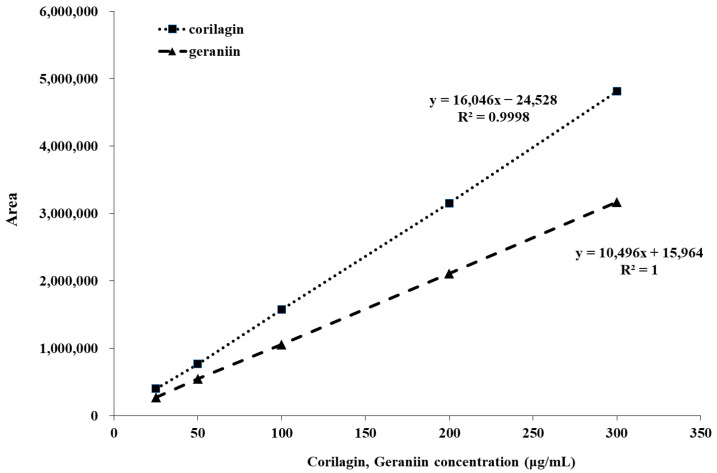
Calibration curves presenting corilagin and geraniin obtained by HPLC–UV at 270 nm in the concentration range of 25–300 µg/mL.

The linearity of the detector response for CG and GR was assessed across the concentration range of 25–300 µg/mL. Calibration curves exhibited excellent linearity, with correlation coefficients (R^2^) greater than 0.995 for both analytes (Figure 7). These results meet the acceptance criterion for linearity, which requires an R^2^ value of at least 0.99 [27].

#### 2.2.3. Precision and Accuracy Results

The results for repeatability (intra-day) and reproducibility (inter-day) are summarized in Table 2 and Table 3. Method precision was evaluated over three consecutive days, with results expressed as relative standard deviation (RSD). The intra-day precision showed RSD values of 0.75% for CG and 1.19% for GR. Inter-day precision yielded similarly low RSD values of 0.77% for CG and 0.98% for GR, confirming good reproducibility.

Accuracy was assessed by spiking the *G. wilfordii* extract with standard solutions to obtain CG concentrations of 150–210 µg/mL and GR concentrations of 200–280 µg/mL. The recovery values ranged from 99.51–100.74% for CG and 99.62–101.23% for GR, confirming the high analytical accuracy of the method (Table 4).

#### 2.2.4. Limits of Detection and Quantification Results

The limit of detection (LOD) is defined as the lowest concentration of an analyte that can be reliably detected, whereas the limit of quantification (LOQ) represents the lowest concentration that can be determined with acceptable accuracy and precision. The LOD values were 0.65 µg/mL for CG and 0.81 µg/mL for GR, while the corresponding LOQ values were 1.97 µg/mL for CG and 2.46 µg/mL for GR (Table 5). These low values indicate that the proposed method provides adequate sensitivity for the quantitative determination of CG and GR in the *G. wilfordii* extract.

Although the total run time of the method is relatively long, this limitation is primarily attributed to the use of a conventional HPLC system and the long analytical column required to achieve baseline separation. Further reduction in analysis time could be achieved by adapting the method to ultra-performance liquid chromatography (UPLC), which employs columns with smaller particle sizes and internal diameters. Previous studies have shown that UPLC can significantly shorten analysis time while maintaining separation efficiency and selectivity [28]. Therefore, the present method represents a suitable platform for future adaptation to UPLC-based analysis.

## 3. Materials and Methods

### 3.1. Reagents and Chemicals

All reagents and chemicals used were of HPLC or analytical grade, including acetonitrile, methanol, and phosphoric acid (DAEJUNG, Siheung, Republic of Korea). Corilagin (≥98% purity, BF-C1016) and geraniin (≥95% purity, BF-G4005) were obtained from BIOFRON Inc. (La Mirada, CA, USA). PVDF membrane filters (0.45 µm) were purchased from Hyundai Micro (Seoul, Republic of Korea). All reagents and chemicals used in this study were of HPLC or analytical grade, including acetonitrile, methanol, and phosphoric acid (DAEJUNG, Siheung, Republic of Korea). Corilagin (≥98% purity, BF-C1016) and geraniin (≥95% purity, BF-G4005) were obtained from BIOFRON Inc. (La Mirada, CA, USA). Polyvinylidene fluoride (PVDF) membrane filters (0.45 µm) were purchased from Hyundai Micro (Seoul, Republic of Korea).

### 3.2. Preparation of G. wilfordii Extract

The whole dried plant of *G. wilfordii* was obtained from the Yeongcheon Herbal Market (Yeongcheon, Republic of Korea). Extraction was performed at 60 °C for 6 h using 30% (*v*/*v*) ethanol at a solid-to-solvent ratio of 10% (*w*/*v*), based on the dry weight of *G. wilfordii* [29]. The extract was centrifuged at 3000 rpm for 20 min, and the supernatant was filtered through filter paper (Whatman No. 6, Whatman International, Maidstone, UK). The filtrate was concentrated under reduced pressure using a rotary evaporator (Eyela SB-1000, Tokyo, Japan), and the resulting concentrate was subsequently freeze-dried using a freeze dryer (FD 8508, Ilshin BioBase, Co., Ltd., Dongducheon-si, Gyeonggi-do, Republic of Korea) to obtain a powdered extract.

A total of 50 g of dried plant material was used for extraction, yielding 6.53 g of the final powdered extract, corresponding to an extraction yield of 13.06% (*w*/*w*). The freeze-dried extract was obtained as a deep-brown, fine powder.

### 3.3. Preparation of Standard Solution

Stock standard solutions of corilagin (CG) and geraniin (GR) were prepared individually at a concentration of 1000 µg/mL by dissolving 10 mg of each compound in 10 mL of 20% (*v*/*v*) methanol. Working standard solutions of CG and GR at concentrations of 25, 50, 100, 200, and 300 µg/mL were prepared by serial dilution of the corresponding stock solutions.

### 3.4. Preparation of Sample Solution

Freeze-dried powder (50 mg) of *G. wilfordii* extract was accurately weighed and transferred into a 10 mL volumetric flask. The volume was adjusted with 20% (*v*/*v*) methanol, and the solution was filtered through a PVDF membrane filter (0.45 µm) prior to HPLC analysis.

### 3.5. HPLC Instruments

HPLC analysis was conducted using a Shimadzu LC-20A system (Shimadzu, Kyoto, Japan) equipped with a binary pump and a photodiode array (PDA) detector (SPD-M20A, Shimadzu). Chromatographic separations were evaluated using several reversed-phase C18 columns, including an XDB-C18 column (150 mm × 4.6 mm, 3.5 µm, Agilent, Santa Clara, CA, USA) and Polaris 3 C18-A columns (150 mm × 4.6 mm, 3 µm and 250 mm × 4.6 mm, 3 µm, Agilent). Data acquisition and processing were performed using LabSolutions software version 5.92 (Shimadzu).

For method optimization, various mobile-phase compositions were systematically evaluated, and the condition providing optimal resolution and quantitative performance was selected. The optimized mobile phase consisted of solvent A (water containing 0.2% formic acid, *v*/*v*) and solvent B (acetonitrile) and was operated under isocratic conditions with acetonitrile contents ranging from 8% to 12% (*v*/*v*). The final analytical parameters were as follows: detection wavelength, 270 nm; flow rate, 1.0 mL/min; injection volume, 10 µL; and column temperature, 30 °C.

### 3.6. Method Validation

The analytical method for the simultaneous determination of CG and GR was validated in accordance with the AOAC guidelines [30]. The validation parameters included specificity, linearity, accuracy, precision, limit of detection (LOD), and limit of quantification (LOQ).

#### 3.6.1. Specificity

Specificity was evaluated to confirm the ability of the method to clearly distinguish CG and GR from other components present in the sample matrix. Specificity was assessed by comparing chromatograms obtained from standard solutions and *G. wilfordii* extract samples, as well as by analyzing procedural blanks. No interfering peaks were observed at the retention times of CG and GR. In addition, the UV spectra of CG and GR in standard and sample solutions were identical, further confirming the specificity of the method.

#### 3.6.2. Linearity

Linearity was evaluated by analyzing five concentration levels of mixed standard solutions and constructing calibration curves by plotting peak area at 270 nm against the corresponding concentrations. Each concentration was analyzed in triplicate. The method exhibited excellent linearity for both CG and GR over the concentration range of 25–300 µg/mL, with correlation coefficients (R^2^) greater than 0.995.

#### 3.6.3. Accuracy by Recovery

The accuracy of the method was evaluated through recovery experiments. Known amounts of CG and GR standard solutions were spiked into the *G. wilfordii* extract matrix at three concentration levels. The spiked samples were then prepared and analyzed under the established HPLC conditions. The percentage recovery of each analyte was calculated to assess the accuracy and reliability of the proposed method.

#### 3.6.4. Precision

Precision, which reflects the reproducibility of the analytical method, was evaluated in terms of the relative standard deviation (RSD%). Intra-day precision (repeatability) was assessed by performing five replicate analyses on the same day (n = 5). Inter-day precision (reproducibility) was evaluated by conducting replicate analyses over three consecutive days (n = 3 × 5) using the same HPLC system under identical experimental conditions.

For this evaluation, *G. wilfordii* extract solutions were independently prepared at three concentration levels (5, 10, and 15 mg/mL) by accurately weighing the appropriate amounts of freeze-dried extract and dissolving them in 20% (*v*/*v*) methanol, following the sample preparation procedure described in Section 3.4. Each concentration level was subsequently analyzed using the validated HPLC method to assess both repeatability and reproducibility.

#### 3.6.5. Limit of Detection (LOD) and Limit of Quantification (LOQ)

The limits of detection (LOD) and quantification (LOQ) were determined based on the standard deviation of the response (σ) and the slope (s) of the calibration curve. Calibration curves for CG and GR were constructed over the concentration range of 25–300 µg/mL. In this study, σ was calculated from the residual standard deviation of the calibration curve. The LOD and LOQ were calculated using the equations LOD = 3.3 × σ/s and LOQ = 10 × σ/s, respectively.

## 4. Conclusions

This study developed and validated an isocratic HPLC–UV method for the simultaneous determination of corilagin (CG) and geraniin (GR) in *Geranium wilfordii* extract. The optimized chromatographic conditions—an acetonitrile/water mobile phase (11:89, *v*/*v*), a Polaris 3 C18-A column (250 mm × 4.6 mm, 3 µm), a flow rate of 1.0 mL/min, and UV detection at 270 nm—enabled high-resolution separation, with retention times of 21.166 min for CG and 32.247 min for GR (Rs > 1.5; tailing factor < 2).

The validated method demonstrated excellent analytical performance, including high specificity, good linearity over the concentration range of 25–300 µg/mL (R^2^ > 0.995), satisfactory precision (RSD% < 1.2%), and high accuracy (recovery: 99.51–101.23%). The limits of detection and quantification were 0.65 and 1.97 µg/mL for CG and 0.81 and 2.46 µg/mL for GR, respectively, confirming the suitability of the method for quantitative analysis.

Compared with gradient HPLC and mass spectrometry-based approaches, the proposed isocratic method offers a simplified analytical workflow with improved reproducibility and reduced operational complexity. Overall, this validated isocratic HPLC–UV method provides a robust and practical analytical platform for routine quality control and phytochemical standardization of *G. wilfordii* extract, with potential applicability in pharmaceutical, food, and cosmetic research and development.

## Figures and Tables

**Table 1:** HPLC conditions for the quantitative analysis of corilagin and geraniin.

Parameter	Conditions
Instrument	SHIMADZU LC-20A (Shimadzu, Kyoto, Japan)
Detector	SHIMADZU PDA Detector (Shimadzu, Kyoto, Japan) (SPD-M20A, wavelength at 270 nm)
Column	Polaris 3 C18-A 250 mm × 4.6 mm × 3 µm (Agilent, Santa Clara, CA, USA)
Mobile phase	(A) 0.2% formic acid in water 89% (B) Acetonitrile 11%
Run time	40 min
Flow rate	1.0 mL/min
Injection volume	10 µL
Column temperature	30 °C

**Table 2:** The intra-day precision (repeatability) results of corilagin and geraniin in the *Geranium wilfordii* extract (*n* = 5).

Compound	Sample Concentration (mg/mL)	Contents (mg/g)	RSD (%)
Corilagin	5	19.22 ± 0.11	0.55
	10	19.06 ± 0.04	0.20
	15	19.01 ± 0.03	0.16
Geraniin	5	23.88 ± 0.21	0.90
	10	24.22 ± 0.22	0.89
	15	24.32 ± 0.26	1.09

Relative Standard Deviation (RSD%): 100 × $\sqrt{\frac{1}{N-1}\sum_{i=1}^{N}{\left(x_{i}-\bar{x}\right)}^{2}}$/$\bar{x}.$

**Table 3:** The inter-day precision (reproducibility) results of corilagin and geraniin in the *Geranium wilfordii* extract (*n* = 5).

Compound	Repetition	Contents (mg/g)	RSD (%)
Corilagin	A	19.26 ± 0.13	0.66
	B	19.11 ± 0.15	0.80
	C	19.21 ± 0.15	0.81
Geraniin	A	24.18 ± 0.09	0.39
	B	24.13 ± 0.38	1.56
	C	23.96 ± 0.15	0.63

Relative Standard Deviation (RSD%): 100 × $\sqrt{\frac{1}{N-1}\sum_{i=1}^{N}{\left(x_{i}-\bar{x}\right)}^{2}}$/$\bar{x}.$

**Table 4:** Accuracy (% recovery) of HPLC analysis for corilagin and geraniin quantification (*n* = 3).

Compound	Amount Added (µg/mL)	Amount Found (µg/mL)	Recovery (%)	RSD (%)
Corilagin	150	149.64 ± 0.27	99.76 ± 0.18	0.18
	180	179.85 ± 0.63	99.92 ± 0.35	0.35
	210	210.97 ± 0.66	100.46 ± 0.31	0.31
Geraniin	200	199.82 ± 0.26	99.91 ± 0.26	0.26
	240	241.56 ± 0.44	100.65 ± 0.44	0.44
	280	279.93 ± 0.17	99.97 ± 0.44	0.17

Relative Standard Deviation (RSD%): 100 × $\sqrt{\frac{1}{N-1}\sum_{i=1}^{N}{\left(x_{i}-\bar{x}\right)}^{2}}$/$\bar{x}.$

**Table 5:** HPLC data of limit of quantification and detection of corilagin and geraniin.

Compound	Retention Time (min)	Range (µg/mL)	LOD (µg/mL)	LOQ (µg/mL)
Corilagin	21.180	25–300	0.65	1.97
Geraniin	32.343	25–300	0.81	2.46

LOD: Limit of detection; LOQ: Limit of quantification.

## Data Availability

The original contributions presented in this study are included in the article. Further inquiries can be directed to the corresponding author.
